# The AT1 Receptor Blocker Telmisartan Reduces Intestinal Mucus Thickness in Obese Mice

**DOI:** 10.3389/fphar.2022.815353

**Published:** 2022-03-31

**Authors:** Laura Nickel, Annika Sünderhauf, Elias Rawish, Ines Stölting, Stefanie Derer, Christoph Thorns, Urte Matschl, Alaa Othman, Christian Sina, Walter Raasch

**Affiliations:** ^1^ Institute of Experimental and Clinical Pharmacology and Toxicology, University of Lübeck, Lübeck, Germany; ^2^ DZHK (German Centre for Cardiovascular Research), Partner Site Hamburg/Kiel/Lübeck, Lübeck, Germany; ^3^ Division of Nutritional Medicine, University Hospital Schleswig-Holstein, Lübeck, Germany; ^4^ Institute of Pathology, University of Lübeck, Lübeck, Germany; ^5^ Department Virus Immunology, Heinrich Pette Institute, Leibniz Institute for Experimental Virology, Hamburg, Germany; ^6^ CBBM (Centre of Brain, Behaviour and Metabolism), University of Lübeck, Lübeck, Germany; ^7^ Institute for Clinical Chemistry, University Hospital Zürich, Zürich, Germany

**Keywords:** AT1 receptor antagonist, mucus thickness, obesity, necroptosis, MUC2 gene expression, telmisartan, goblet cell, diet induced obesity (DIO)

## Abstract

The angiotensin II (type 1) (AT_1_) receptor blocker telmisartan (TEL) is beneficial for the treatment of individuals suffering from metabolic syndrome. As we have shown that TEL has an impact on gut microbiota, we investigated here whether TEL influences gut barrier function. C57BL/6N mice were fed with chow or high-fat diet (HFD) and treated with vehicle or TEL (8 mg/kg/day). Mucus thickness was determined by immunohistochemistry. Periodic Acid-Schiff staining allowed the number of goblet cells to be counted. Using western blots, qPCR, and immunohistochemistry, factors related to mucus biosynthesis (*Muc2, St6galnac*), proliferation (*Ki-67*), or necroptosis (*Rip3*) were measured. The influence on cell viability was determined *in vitro* by using losartan, as the water solubility of TEL was too low for *in vitro* experiments. Upon HFD, mice developed obesity as well as leptin and insulin resistance, which were prevented by TEL. Mucus thickness upon HFD-feeding was diminished. Independent of feeding, TEL additionally reduced mucus thickness. Numbers of goblet cells were not affected by HFD-feeding and TEL. *St6galnac* expression was increased by TEL. *Rip3* was increased in TEL-treated and HFD-fed mice, while *Ki-67* decreased. Cell viability was diminished by using >1 mM losartan. The anti-obese effect of TEL was associated with a decrease in mucus thickness, which was likely not related to a lower expression of *Muc2* and goblet cells. A decrease in *Ki-67* and increase in *Rip3* indicates lower cell proliferation and increased necroptosis upon TEL. However, direct cell toxic effects are ruled out, as *in vivo* concentrations are lower than 1 mM.

## Introduction

Obesity is an important global health problem and associated with a large decrease in life expectancy due to a higher risk for cardiometabolic complications (Engin, 2017). Effective pharmacotherapy achieving at least 5% weight loss was indeed observed in the majority of patients treated with lorcaserin, orlistat, or phentermine/topiramate, but no obesity medication reduced cardiovascular morbidity or mortality (Yanovski and Yanovski, 2014). Thus, new pharmacological strategies are needed.

The renin‒angiotensin system (RAS) is well-known to be involved in cardiovascular and metabolic regulation. Angiotensin II (AngII) increases blood pressure, impairs metabolic functions such as glucose control, and promotes the development of atherosclerosis. Thus, AngII receptor (type 1) blockers (ARBs) such as telmisartan (TEL) and losartan (LOS) are well-established in the treatment of hypertension and heart failure, particularly with regard to their cardiometabolic benefits (Michel et al., 2016). Beyond these beneficial actions, ARBs have been demonstrated to preventively and curatively lower obesity in rodents (Muller-Fielitz et al., 2011; Miesel et al., 2012; Muller-Fielitz et al., 2012; Muller-Fielitz et al., 2014; Muller-Fielitz et al., 2015; Schuchard et al., 2015; Schuster et al., 2018; Winkler et al., 2018; Gustaityte et al., 2019; Rawish et al., 2020; Huber et al., 2021) and humans (Kintscher et al., 2007). The anti-obese potency of ARBs occurs mainly after high dosages and is independent of their ability to reduce blood pressure (Muller-Fielitz et al., 2011; Muller-Fielitz et al., 2014). ARB-induced weight loss correlates with a reduction of energy intake, fat mass, and size of adipocytes (Miesel et al., 2012; Muller-Fielitz et al., 2012). Although the underlying mechanism remains a matter of debate, it was found that leptin-related (Muller-Fielitz et al., 2011; Muller-Fielitz et al., 2012; Schuster et al., 2018) and other brain-related mechanisms (Winkler et al., 2016; Rawish et al., 2020; Huber et al., 2021) are involved in ARB-induced regulation of energy homeostasis. Addressing brain-related mechanisms, we have repeatedly shown that obesity-induced leptin resistance is normalized by TEL, as leptin can once again cross the blood‒brain barrier (Muller-Fielitz et al., 2011; Muller-Fielitz et al., 2012; Schuster et al., 2018). We moreover found in 2020 that TEL has anti-obese efficacy and prevents lipid accumulation and lipotoxicity, which is accompanied by an anti-inflammatory effect in the murine hypothalamus, thus also supporting the notion that a brain-related mechanism is involved in TEL-induced weight loss (Rawish et al., 2020). We very recently showed that TEL protects against neurovascular unit impairments in a diet-induced obesity setting, which may play a role in preventing obesity-related cognitive deficits, as we demonstrated that TEL treatment normalized high-fat diet-induced reduction of cerebral blood flow and prevented diet-induced anxiety-like behavior and that TEL affects cellular senescence and string vessel formation in obesity (Huber et al., 2021). In addition to these putative mechanisms, an angiotensin-converting enzyme 2 (ACE2)/Ang (1–7)/Mas axis-related mechanism (Blanke et al., 2015; Schuchard et al., 2015; Dapper et al., 2019) is also involved in ARB-induced weight loss, while the pleiotropic potency of ARBs to stimulate peroxisome proliferator-activated receptor gamma (PPARγ) (Muller-Fielitz et al., 2012; Schuster et al., 2018) and the potency of ARBs to lower stress (Gustaityte et al., 2019) play only a minor or no role.

In the context of gut-dependent parameters, obesity is known to be related to an increased *Firmicutes*/*Bacteroidetes* (*F/B*) ratio (Stephens et al., 2018) and also features a metabolic inflammatory state (Gregor and Hotamisligil, 2011). In response to excess nutrients and energy, bacterial lipopolysaccharide (LPS)-producing bacteria increase in the intestine. Hence, LPS levels increase in plasma and intestine, thereby suggesting that LPS is a triggering factor for low-grade inflammation in obesity (Cani et al., 2007). Further addressing the interaction between the RAS and obesity, we recently identified that the anti-obese action of TEL is attributed to diet-independent alterations in gut microbiota, as we found that: 1) the *F/B* ratio and the abundancies of *Blautia*, *Allobaculum*, and *Parasutterella* were higher in rats that were fed with cafeteria diet (CD) than in rats that were additionally treated with TEL; 2) that enterotype-like clustering analyses, Kleinberg’s hub network scoring, and random forest analyses also indicated that TEL induced a specific signature of gut microbiota; and 3) that in response to stool transfer from TEL-pretreated donor to high-fat fed acceptor mice, body weight gain was slightly attenuated (Beckmann et al., 2021).

Gut bacteria‒epithelial cell interactions are key regulators of epithelial permeability (Allam-Ndoul et al., 2020). Following metabolic endotoxemia, intestinal barrier function may be impaired due to changes in tight junction protein expression or modifications of the passage of potentially harmful bacterial antigens and microorganisms from the intestinal lumen to systemic circulation (Winer et al., 2016). The intestinal mucosa is the innermost layer of the intestinal tract, providing support and mobility to the mucosa. The intestinal mucosa is overlaid with a discontinuous mucus layer that forms a highly organized glycol protein network. Colonic mucus is mainly composed of mucin proteins, especially Mucin 2 (*Muc2*), secreted by specialized epithelial cells known as goblet cells. This gel-like structure acts as a physical barrier, permeable to water and small molecules, limiting direct contact between the contents of the gut lumen and epithelial cells (Maloy and Powrie, 2011; Schroeder, 2019). It also displays antimicrobial properties through the action of secreted antimicrobial peptides, but also represents an important bacterial niche. The thickness and composition of the mucus layer influence the properties of this bacterial niche, whilst the bacteria can also impact the properties of the mucus layer (Bergstrom et al., 2010). Addressing this interaction between mucus composition and gut microbiota, the genera *Parabacteroides* and *Blautia* increased in fecal samples of rats in response to Ang II, while *Ruminococcus* and *Oscillospira* decreased, and moreover, the thickness of the jejunum of small intestine showing tunica muscularis increased, while the numbers of goblet cells and villi length decreased (Sharma et al., 2019). In this regard, the findings on *Blautia* seem particularly interesting, as in our recent paper we were able to show an increase in the abundance of *Blautia* in CD-fed rats and a normalization of *Blautia* when the rats were simultaneously treated with TEL (Beckmann et al., 2021). Also considering the above-mentioned relationships between gut microbiota and intestinal trans-epithelial permeability (Allam-Ndoul et al., 2020), the aim of the present study was to investigate whether TEL influenced mucus itself. Therefore, we determined crypt depths, number of goblet cells, thickness of mucus, and expression of various factors addressing mucus production and its glycosylation, as well as necroptosis indicators in colon samples of mice that were fed for 3 months with standard chow diet or HFD and concurrently treated with TEL or vehicle.

## Methods

### Animals

All animal care and experimental procedures were performed in accordance with the National Institutes of Health (NIH) guidelines on the care and use of laboratory animals and were approved by the animal ethics committee of the local regulatory authority (Ministerium für Energiewende, Landwirtschaft, Umwelt, Natur und Digitalisierung des Landes Schleswig-Holstein, Germany) under application number 109-8/15. The results of all studies involving animals are reported in accordance with Animal Research: Reporting of *In Vivo* Experiments (ARRIVE) guidelines. Male C57BL/6N mice (from Charles River Laboratories, Sulzfeld, Germany) aged 6‒8 weeks were kept in individually ventilated cages in groups of between two and five individuals at 23°C under a 12 h/12 h dark (6 p.m.‒6 a.m.)/light (6 a.m.‒6 p.m.) cycle. All animals were habituated to laboratory conditions for at least 10 days before experiments were started. All animals had free access to water. A total of 48 mice were included in the study.

### Study Protocol

Group size was assessed by power analysis (alpha value = 0.017, power 80%) to be at least n = 13 in each group when expecting a weight difference of 8 ± 6 g after TEL treatment. Two mice died of an unexpected anesthetic accident during magnetic resonance imaging (MRI) analyses. All animals were monitored daily by visual inspection and weighing. Mice received one of the following two diets ad libitum: HFD (EF acc. D12492 [I] mod. from ssniff^®^, Soest, Germany) with 24.0 MJ/kg or normal-fat diet (NFD, EF acc. D12450B mod. from ssniff^®^, Soest, Germany) with 18.0 MJ/kg. Once a day, mice received TEL (8 mg/kg_bw_) or vehicle by oral gavage in a volume of 5 µL per gram body weight (bw) according to previous studies (Schuster et al., 2018; Dapper et al., 2019; Rawish et al., 2020). TEL dosage was recently evaluated (Muller-Fielitz et al., 2012) and has been confirmed in numerous studies both in rats (Miesel et al., 2012; Muller-Fielitz et al., 2014; Muller-Fielitz et al., 2015; Schuchard et al., 2015; Winkler et al., 2016; Gustaityte et al., 2019) and in mice (Schuster et al., 2018; Dapper et al., 2019; Rawish et al., 2020; Huber et al., 2021) to reveal anti-obese effects. For administration, TEL was suspended in 10% gum arabic (Carl Roth GmbH, Karlsruhe, Germany), resulting in a suspension of 1.6 mg/ml TEL. Mice were allocated by block randomization to the following groups: chow and vehicle treatment (chow_VEH_), HFD and vehicle treatment (HFD_VEH_), and HFD or chow and TEL treatment (chow_TEL_, HFD_TEL_) (Supplementary Figure S1). Mice were phenotyped at week 6 with respect to energy expenditure (EE), respiratory exchange rate (RER), locomotion, as well as drinking and feeding behavior using the PhenoMaster SystemTM (TSE, Bad Homburg, Germany) as previously described (Schuster et al., 2018; Dapper et al., 2019; Rawish et al., 2020). Data were analyzed separately for light and dark periods (only considering a core period of 8 h for each period) and averaged for the 5 days. At week 7, insulin sensitivity was determined by performing an insulin tolerance test (ITT) as recently described (Schuster et al., 2018; Dapper et al., 2019; Rawish et al., 2020). At week 10, fat mass was assessed by means of MRI (Philips, Achieva, 1.5 T, 8-channel SENSE) (Schuster et al., 2018; Dapper et al., 2019; Rawish et al., 2020). At week 11, tail blood was withdrawn after a 6-h fasting period to determine plasma lipids. At week 13, mice were sacrificed, whereupon organs and blood were removed. The distal end of the colon was fixed in Carnoy’s solution. For this purpose, the most distal piece of intestine, which still contained feces, was removed. The remaining parts of the intestine were rinsed with ice-cold phosphate buffered saline (PBS) before they were fixed. Other organs were frozen in liquid nitrogen and stored at ‒80°C before further analyses.

### Histology and Immunohistochemistry

The standard tissue paraffin block was sectioned at 4 µm. Sections were mounted on microscope slides and heated at 60°C for 1 h to attach the sections to the slide. Prior to staining, sections were deparaffinized in three changes of xylene and rehydrated through graded concentrations of ethanol. The histological stains were hematoxylin and eosin (H&E) (Gill et al., 1974), periodic acid Schiff (PAS) (McMANUS, 1946), and *Ki-67* staining (Braasch et al., 2011). H&E staining is a primary diagnostic tool used in histology allowing visualization of morphological changes in tissues. PAS staining allows in particular the demonstration of carbohydrate-containing components such as glycoproteins, mucins, and glycogen. *Ki-67* staining provides indirect information about the growth rate of cells. During the cell cycle, *Ki-67* is expressed in the G1, S, G2, and M phases, while resting cells in the G0 phase do not express the *Ki-67* antigen.

H&E and PAS staining were performed in tissue samples of each animal according to established protocols in the routine pathology lab. All H&E images (exemple images are depicted in Supplementary Figure S5) were inspected in a blinded manner by an experienced pathologist for microscopically visible changes in the context of inflammation. PAS images were evaluated using the ZEN 2.3 imaging software (Zeiss, Jena, Germany). Four images per animal (10-fold magnification with an exposure time of 200 ms) were taken, each of the proximal and distal parts of the large intestine. The number of goblet cells within a crypt was counted in 10 crypts per image. Furthermore, the respective crypt depth was measured in these 10 defined crypts per image.

For *Ki-67* staining, large intestine sections were preheated at 56°C, then deparaffinized and rehydrated with ddH_2_O. After endogenous peroxidase blocking with 3% H_2_O_2_ in methanol for 5 min, antigen retrieval took place in pre-warmed 10-mM citrate buffer (pH 6.0) at 95°C. The slides were treated with Triton X-100 0.1% (Sigma-Aldrich, St. Louis, MO, United States) for 10 min, followed by avidin and biotin blocking with egg solution and 5% milk for 10 min each. Further blocking steps took place under serum-free media (Dako Inc., Glostrup, Denmark) for 1‒2 h at room temperature. The slides were thoroughly rinsed with Tris-buffered saline (pH 7.2) between each step. Next, the primary antibody (monoclonal rat anti-*Ki-67* antigen IgG2a, clone TEC-3 [M7249], Dako) was diluted in serum-free media to 1:100 and placed on the slides for 2 h at room temperature. The slides were thoroughly rinsed and the secondary antibody (Rat HRP-Polymer kit [RT517H, Biocare Medical, Concord, CA, United States]) was applied. The slides were stained with the Chromogen-DAB kit (Vector Labs, Burlingame, CA, United States) for 6 min, then rinsed, dehydrated, and the coverslip mounted with solvent S-100 (Braasch et al., 2011). The staining was quantified with ImageJ (version 2.0.0-rc-54/1.51 h) by defining four regions of interest (ROI, 150 × 200 pixels) for each image. The stained cells were counted within these ROIs. Based on the *Ki-67* labeling index (Sasaki et al., 1988), the number of Ki-67-positive cells was given in percent.

Mucin-2 (Muc2) staining was carried out on Carnoy-fixed intestinal tissue. Tissue was double-stained with the DNA dye Hoechst 33,342 (Cambrex IEP, Germany) to visualize nucleated cells, thus allowing better recognition of tissue structures. After Carnoy fixation (for at least 48 h), tissue was rinsed with PBS (2 × 5 min). At 20 min after adding the blocking solution and goat serum, slices were incubated (60 min at 4°C) with the primary antibody (100 µl/slice, anti-α*Muc2*-antibody; rabbit, Santa Cruz Biotechnology, Inc., United States, 1:100 in PBS) and for isotype control anti-rabbit-antibody (100 µl/slice, goat, R&D Systems, Minneapolis, MN, United States, 1:500 in PBS). Next, slices were rinsed with PBS (2 × 5 min) and re-incubated with the secondary antibody (100 µl per slice, 60 min anti-rabbit antibody; goat, 594, Alexa, Life Technologies GmbH, Germany). After rinsing once more (PBS, 2 × 5 min), slices were stained with Hoechst 33,342 (PA-3014) (Cambrex IEP, Deutschland, 1:10,000, 5 min). After a final rinsing step with PBS (2 × 5 min), slices were covered with Mowiol and dried for 30 min. The evaluation of *Muc2* immunofluorescence staining was performed using the ZEN 2.3 imaging software (Zeiss, Germany). An average of four images per animal were taken (10x magnification with an exposure time of 200 ms). An average of 10 measurement points were identified per image, which, distributed over the length of the image, had the same distance. The thickness of the mucus layer was measured in micron. This resulted in approximately 40 measuring points per animal. The groups were blinded for the evaluation (Supplementary Figure S4).

### Quantitative Polymerase Chain Reaction

Using quantitative polymerase chain reaction (qPCR), the expression of various RAAS components (AT_1A_, AT_1B_, AT_2_ receptors), factors addressing mucus production and its glycosylation (*Muc2, Gcnt2, Gcnt3, C1galt1, St6galnac, Atoh1, Spdef1*), cell proliferation (*Ki-67*), and necroptosis (*Rip3*) indicator were determined in colonic samples from each mouse of each test group. Tissue samples were homogenized and total RNA was isolated using a commercial kit according the manufacturer’s instructions (innuPrep RNA Mini-Kit, Analytic Jena, Germany). The mRNA concentration was measured using the NanoDrop 2000 system (Thermo Fisher Scientific Inc., United States). The samples were stored at ‒20°C until cDNA synthesis. Also following the manufacturer’s instruction, the commercially available RevertAid H Minus Reverse Transcriptase kit (Thermo Fisher Scientific Inc., United States) was used to transcribe mRNA into cDNA. The cDNA was amplified using the Maxima SYBR Green qPCR Master Mix (Thermo Fisher Scientific Inc., United States) with primers specific to the expression products (Table 1, biomers.net GmbH, Germany) using the ABI PRISM^®^ 7,000 Sequence Detection System (Life Technologies Corporation, Germany). The expression of *β-actin* was determined for each sample and all results were related to the number of cDNA copies of β-actin. To check the size, quantity, and quality of the product, agarose gel electrophoresis was carried out on the qPCR products according to standard methods.

**TABLE 1:** Primer sequences.

Primer	Forward	Backward
ß-actin	5′-GAT GCT CCC CGG GCT GTA TT-3′	5′-GGG GTA CTT CAG GGT CAG GA-3′
AT_1A_	5‘-TTA GCA ATG GAG ACC CAC GT-3‘	5‘-CCA CTA ACT GGC ATT GTT TGG-3‘
AT_1B_	5‘-CTT CAA TGC CCT CCC AAT TCT-3‘	5‘-TGC TTC CTT GTC CCT TGG AA-3‘
AT_2_	5’-TTT AAG GAG TGC ATG CGG GA-3′	5′-AAA GGA CGG CTG CTG GTA AT-3′
*Muc2*	5′-GCT GAC GAG TGG TTG GTG AAT G-3′	5′-GCT GAC GAG TGG TTG GTG AAT G-3′
*Ki-67*	5′-CCT GCC CGA CCC TAC AAA AT-3′	5′-TTG CTC ACA CTC GAT GCA GT-3′
*Rip3*	5′-AGC TTT GGG ATC CTC GTG TG-3′	5′-TGT CAG TGG AGG ACG ACT CT-3′
*Gcht2*	5′-AGT GGA TGG ACA TGG AAG CG-3‘	5’-CTC GAA GCC TCA GTT CCA GG-3′
*C1galt1*	5′-GGA GCG GGC TAT GTC CTA AG-3‘	5’-GAA CAG CAT CCA GGA CCC TC-3′
*St6galnac*	5′-ACA GGA GCA GTG TCA ACA AG-3′	5′-ACA GGA GCA GTG TCA ACA AG-3′
*Gcth3*	5′-CTG TAC AAG ACC CTG AAG C-3′	5′-GAC ATT GGG GAA GCA TGA C-3′
Atoh1	5′-GTG GGG TTG TAG TGG ACG AG-3‘	5’- GTT​GCT​CTC​CGA​CAT​TGG​G-3‘
*Klf4*	5’-GGG AAG GGA GAA GAC ACT GC-3‘	5’-GGT GGG TTA GCG AGT TGG A-3‘
*Spdef*	5’-GGA GAA GGC AGC ATC AGG A-3′	5′-CCA GGG TCT GCT GTA ATG T-3′

### Western Blots

Western blot analyses were performed in a subgroup of n = 3 per experimental group, as only 12 samples could be analyzed in one blot. Proteins were extracted from tissue samples applying a denaturing lysis buffer (DLB). Frozen biopsies were first crushed with a pestle and then resuspended in 150–200 μl DLB buffer containing 2% protease inhibitor and 1% phosphatase inhibitor II and III each. For further cell lysis, samples were first heated at 100°C for 5 min, shortly cooled on ice followed by two times ultrasound sonication for 20 s. Finally, proteins were separated from cell debris by centrifugation for 15 min at 12,000 x *g* at 4°C. Protein was quantified using Roti®Quant assay (Carl Roth GmbH + Co. KG, Germany). Optical density (OD) was measured at 490 nm against a reference wavelength of 690 nm on a microplate reader, and concentrations were calculated using a bovine serum albumin (BSA) standard curve. After blotting, the samples were transferred onto polyvinylidene fluoride (PVDF) membranes, and the unspecific binding sites were blocked with 5% non-fat milk in Tween-TBS (T-TBS, 0.1% Tween 20) for 1 h at room temperature (RT). Primary antibodies (Table 2, in 5% non-fat milk) were added and incubated at 4°C on a shaker overnight. Following a washing step with T-TBS (2 × 20 min), the secondary antibody (rabbit, Cell Signaling, United States, 1:4,000 in 5% non-fat milk) was added and incubated on a shaker for 1 h and then washed with T-TBS (2 × 20 min). Horse radish polymerase (HRP, Merck KGaA, Germany) solution was evenly distributed on membranes, and chemiluminescence was detected under ultraviolet (UV) light in the ChemiDocTM XRS + imaging system using the ImageLabTM software. Intensity of chemiluminescence was quantified via the Fiji plugin of the ImageJ software (version 2.0.0-rc-54/1.51 h), and expression levels of proteins of interest were normalized to housekeeper proteins.

**TABLE 2:** Primary antibodies.

Primary antibodies	Company
β-Actin (rabbit, 1:1,000 in 5% skim milk, #4967)	Cell Signaling Technology, Inc., United States
Akt (rabbit, 1:1,000 in 5% BSA, #9272)	Cell Signaling Technology, Inc., United States
pAkt ((Thr308), rabbit, 1:500 in 5% BSA, #9257)	Cell Signaling Technology, Inc., United States
Erk ((137F5), rabbit, 1:1,000 in 5% BSA, #4695)	Cell Signaling Technology, Inc., United States
pErk ((Thr202/Tyr204) (D13.14.4E) XP, rabbit, 1:500 in 5 %BSA, #4379)	Cell Signaling Technology, Inc., United States
*Rip3* ((H-207), rabbit, 1:200 in 5% % skim milk, #sc-7881)	Bio-Rad (Formerly SantaCruz), United States

### Cell Culture Experiments

To determine the cell viability of mucus-producing human cells (HT29-MTX, Sigma Aldrich, United States) and murine small intestinal epithelial cells (IEC-1, Sigma Aldrich, United States) in the presence of losartan or enalaprilat. Given that TEL has limited water solubility and thus is not or only partially suitable for *in vitro* experiments, we instead used the AT1 antagonist losartan, which has sufficient water solubility. We used enalaprilat as a control to determine whether a potential influence on cell viability was directly attributable to an AT1-dependent mechanism or indirectly also to the availability of AngII. 4×10^4^ HT29-MTX or 5 × 10^3^ IEC-1 cells were seeded in each well of a 96-well plate and cultivated in culture media (DMEM [+] 4.5 g/l D-Glucose [+] L-Glutamine [−] Sodium Pyruvate, Thermo Fisher Scientific Inc., United States) at 37°C, 5% CO_2_, and 95% humidity. Medium (100 μl/well) was renewed daily and cell growth was checked under the microscope. Cells were incubated with losartan (Merck & Co., Inc. United States, 0.08–50 mM dissolved in glucose‒Krebs Ringer phosphate [KRP] buffer [KRP/11 mM glucose]), enalaprilat (Merck & Co., Inc. United States, 0.125–2 mM dissolved in glucose‒KRP buffer [KRP/11 mM glucose]), medium (serving as positive controls), or TritonX100 (Merck KGaA, Germany, 5% v/v, serving as death controls, n = 5 each condition) at day 8 (HT29-MTX cells) or day 3 (IEC-1 cells). After incubation for 24 h, the MTS assay (CellTiter 96^®^ AQ_ueous_ One Solution cell proliferation assay, Promega, Walldorf, Germany) was carried out according to the manufacturer’s instructions to determine cell viability.

### Biochemical Analysis

Blood glucose was measured using a commercial glucose sensor (Ascensia Elite XL and Elite Sensor, Bayer Vital GmbH, Leverkusen, Germany). Plasma concentrations of diverse adipocytokines were measured using commercially available microsphere-based multiplexing systems in 10 µl of sample by immunosorbent assays according to the manufacturer’s instructions (Milliplex mouse metabolic magnetic bead panel kit MADKMAG-71K-07.mouse and MHSTCMAG-70K-13 mouse high-sensitivity equipment; both from Merck/Millipore, Darmstadt, Germany) (Rawish et al., 2020). The triglyceride and ceramide levels in plasma were analyzed by liquid chromatography-mass spectrometry (LC-MS) according to other published methods (Othman et al., 2012).

### Calculations and Statistics

Data and statistical analysis complied with the recommendations for experimental design and analysis in pharmacology. Food intake was determined by the Phenomaster System and consumed energy was calculated using the energy density of the special diets (24 kJ/g and 18 kJ/g). In order to quantify the total effect over the observation period in response to ITT, areas under the curves (AUC) were calculated for each individual animal based on their plasma glucose delta values. Maximal glucose decline during ITT was also calculated considering delta values. Half-life of glucose decline after insulin exposure was calculated after ln transformation of the glucose concentrations and by determining the slopes of linear regression lines. Correlation analyses were performed by two-tailed Pearson test (GraphPad Prism 8, La Jolla, CA, United States). Dose concentration curves for cell viability were generated and IC_50_ values were calculated by non-linear fitting following log transformation using GraphPad Prism 8 considering the values after correction by the TritonX values that served as dead control. Statistical evaluations were performed using GraphPad Prism 8. All data were checked for outliers and tested for Gaussian distribution by performing D'Agostino&Pearson omnibus normality tests and variance homogeneity. Strain differences were evaluated by two-way or three-way ANOVA (analysis of variance), followed by Sidak’s multiple comparisons test post-test to prove treatment effects. A two-way or three-way ANOVA was only performed when Gaussian distribution and variance homogeneity were detected. Sidak’s multiple comparisons test was only performed if *p* was <0.05. Welch’s correction for ANOVA was considered when variance inhomogeneity between groups was detected. Kruskal-Wallis test with Dunn’s multiple comparison test was used when values were not distributed in a Gaussian fashion. Differences in cell viability were determined by Kruskal-Wallis test followed by Dunn’s multiple comparisons test. In graphs and tables, data are expressed as means ± standard deviation (SD) with the exception of Supplementary Figure S3, in which the data have been presented as means ± standard error (SEM) for clarity.

## Results

### Growth

**FIGURE 1:**
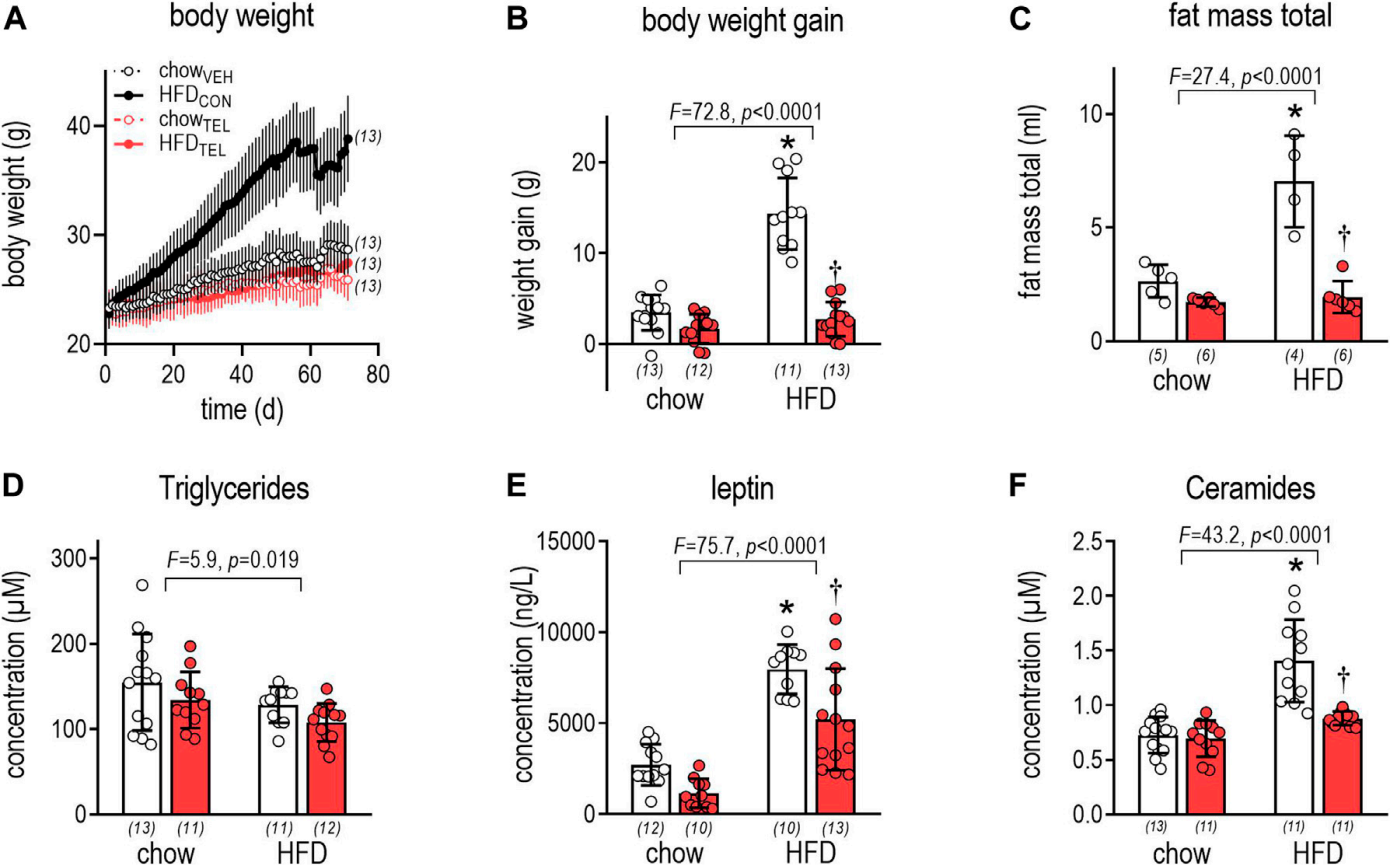
Growth of mice that were fed either with chow or high fat diet (HFD). Mice were treated with TEL (●, 8 mg/kg/day red bars) or vehicle treatment open bars (O). **(A)**: time-dependent increase in body weight (for results of three-way of ANOVA see Supplementary Table S1); **(B)** weight gain within the experimental period of 12 weeks (two-way ANOVA; TEL: *F* = 90.8, *p* < 0.0001, interaction; *F* = 49.3, *p* < 0.0001); **(C)** total fat mass after 10 weeks of treatment (two-way ANOVA; TEL: *F* = 46.5, *p* < 0.0001, interaction; *F* = 22.2, *p* = 0.0002); **(D)** TG plasma concentrations after 12 weeks (two-way ANOVA; TEL: *F* = 3.6, *p* = 0.063, interaction; *F* = 0.1, *p* = 0.999); **(E)** plasma leptin concentration at the end of the study (two-way ANOVA; TEL: *F* = 16.4, *p* = 0.0002, interaction; *F* = 1.3, *p* = 0.27); **(F)** plasma ceramide concentrations after 12 weeks (two-way ANOVA; TEL: *F* = 18.1, *p* = 0.0001, interaction; *F* = 14.1, *p* = 0.0005). Means ± SD, the individual group size is indicated below the bars in brackets. *p < 0.05 vs. chow_VEH_, †*p* < 0.05 vs. HFD_VEH_.

Time-dependent increase of body weight was significantly higher in response to HFD- than to chow-feeding (Figure 1A, Supplementary Table S2). Even though organ weights (left ventricle, liver, kidney) also increased in response to HFD, the higher weight gain (Figure 1B) and particularly higher fat mass (Figure 1C, Supplementary Figures S2A–C) in HFD- than in chow-fed mice clearly indicate that these mice became obese. Development of obesity is suggested to be related to lower energy expenditure rather than to higher energy intake (Table 3, Supplementary Figure S2, Supplementary Table S1). Reduced respiratory ratios in HFD- compared to chow-fed mice indicate an increase in fat burning (Table 2, Supplementary Figure S3, Supplementary Table S1). Attributed to development of obesity, plasma levels of leptin and ceramides increased, while that of triglycerides (TG) decreased (Figures 1D–F). However, development of obesity was prevented despite HFD feeding when mice were simultaneously treated with TEL. This became visible by the normalization of weight gain and fat mass, as well as of plasminogen activator inhibitor (PAI), leptin, and ceramide plasma levels (Figure 1, Supplementary Table S1). This anti-obese effect of TEL was related to a reduced energy intake rather than to an increase in energy expenditure (Table 1). In contrast to HFD feeding, TEL treatment did not alter any growth parameters when mice received standard chow diet (Figure 1, Supplementary Figure S2, Table 1, Supplementary Table S1). Although increased water intake in response to intravenous AngII administration was blocked by intracerebroventricular AT1 receptor blockade (Höhle et al., 1995), drinking responses after TEL were not reduced in this study and were in fact increased (Table 2; Supplementary Figure S3). A TEL-induced increase in water intake of this kind was also found in our recent studies in rats (Muller-Fielitz et al., 2014; Schuchard et al., 2015; Winer et al., 2016) and mice (Schuster et al., 2018; Dapper et al., 2019; Rawish et al., 2020) and has been discussed as possibly having limited cerebral bioavailability. However, there is also clear evidence for cerebral effects after oral ARB administration (see comments in the Introduction).

**TABLE 3:** Energy expenditure, energy intake, drinking, respiratory ratio and locomotion during dark periods of chow and HFD-fed mice which were treated with vehicle or TEL. 2-way ANOVA was calculated considering diet and treatment followed by Sidaks multiple comparisons test. means ± SD, *p < 0.05 vs. chow_VEH_, †*p* < 0.05 vs. corresponding vehicle treatment.

	chow_VEH_ (n = 12)	chow_TEL_ (n = 11)	HFD_VEH_ (n = 12)	HFD_TEL_ (n = 12)	
Energy expenditure (kcal/h/kg_bw_)	30.4 ± 1.1	31.1 ± 1.6	25.1 ± 0.9*	30.2 ± 1.4†	*F* ^diet^ = 6.2, *p* ^diet^ = 0.016
					*F* ^TEL^ = 5.6, *p* ^TEL^ = 0.023
					*F* ^interaction^ = 2.7, *p* ^interaction^ = 0.106
RER	1.00 ± 0.01	0.99 ± 0.01	0.77 ± 0.01*	0.78 ± 0.01	*F* ^diet^ = 1,479, *p* ^diet^<0.0001
					*F* ^TEL^ = 0.1, *p* ^TEL^ = 0.754
					*F* ^interaction^ = 4.3, *p* ^interaction^ = 0.044
Energy intake (kJ)	56.9 ± 2.1	52.4 ± 1.7	41.7 ± 4.0*	33.4 ± 1.3†	*F* ^diet^ = 58.18, *p* ^diet^<0.0001
					*F* ^TEL^ = 8.2, *p* ^TEL^ = 0.007
					*F* ^interaction^ = 0.7, *p* ^interaction^ = 0.414
Locomotion (n)	44.8 ± 6.7	33.2 ± 8.0	24.8 ± 5.3*	59.3 ± 7.7†	*F* ^diet^ = 0.2, *p* ^diet^ = 0.667
					*F* ^TEL^ = 2.7, *p* ^TEL^ = 0.109
					*F* ^interaction^ = 10.8, *p* ^interaction^ = 0.02
drinking (ml)	1.9 ± 0.1	2.9 ± 0.1†	1.1 ± 0.2*	2.7 ± 0.1†	*F* ^diet^ = 10.4, *p* ^diet^ = 0.002
					*F* ^TEL^ = 71.4, *p* ^TEL^<0.0001
					*F* ^interaction^ = 4.2, *p* ^interaction^ = 0.047

**FIGURE 2:**
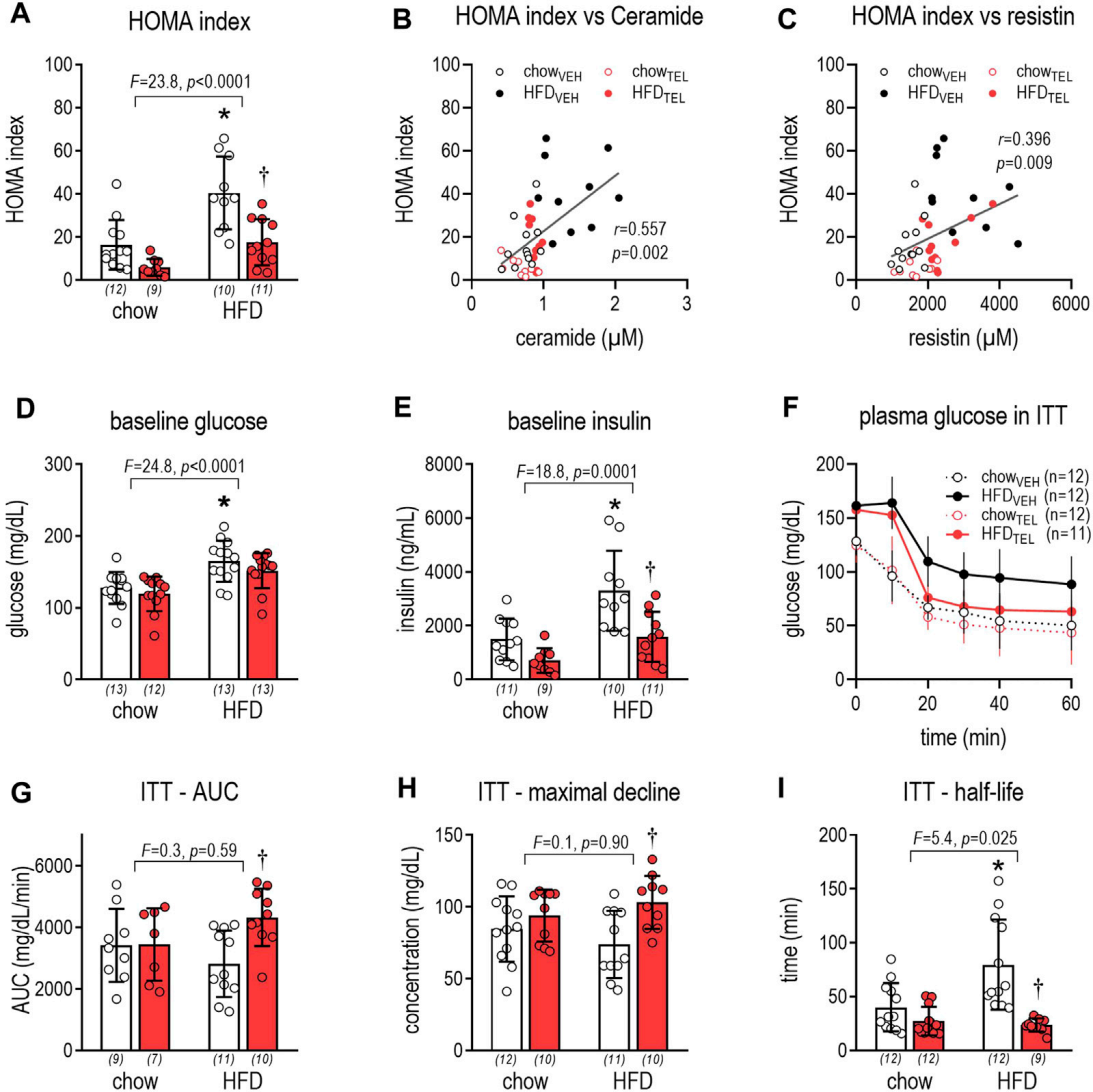
Glucose control of mice that were fed either with chow or high fat diet (HFD). Mice were treated with TEL (●, 8 mg/kg/day red bars) or vehicle treatment open bars (O). **(A)** HOMA index after 12 weeks of treatment (two-way ANOVA; TEL: *F* = 20.1, *p* < 0.0001, interaction; *F* = 2.9, *p* = 0.097); **(B)** correlation between HOMA index and plasma ceramide (Pearson; r = 0.557, *p* = 0.0002); **(C)** correlation between HOMA index and plasma resistin (Pearson; r = 0.395, *p* = 0.0095); **(D)** baseline glucose after 12 weeks of treatment (two-way ANOVA; TEL: *F* = 2.4, *p* = 0.127, interaction; *F* = 0.12, *p* = 0.733); **(E)** baseline insulin concentration after 12 weeks of treatment (two-way ANOVA; TEL: *F* = 16.3, *p* = 0.0003, interaction; *F* = 2.2, *p* = 0.14); **(F)** change in plasma glucose in ITT; **(G)** AUC of plasma glucose levels during ITT (two-way ANOVA; TEL: *F* = 5.2, *p* = 0.028, interaction; *F* = 3.7, *p* = 0.062); **(H)** maximal glucose decline during ITT (two-way ANOVA; TEL: *F* = 9.1, *p* = 0.004, interaction; *F* = 2.4, *p* = 0.127); **(I)** half-life of glucose decline after insulin exposure (two-way ANOVA; TEL: *F* = 19.9, *p* < 0.0001, interaction; *F* = 7.9, *p* = 0.008). Means ± SD, the individual group size is indicated below the bars in brackets.*p < 0.05 vs. chow_VEH_, †*p* < 0.05 vs. HFD_VEH_.

Due to HFD feeding, glucose control deteriorated as non-fasting glucose and insulin levels, as well as the homeostasis model assessment (HOMA) index, increased (Figures 2A,D,E). Moreover, longer half-life in ITT in HFD- than in chow-fed mice indicates that glucose utilization in response to insulin challenge was reduced (Figures 2F,I). TEL treatment effectively prevented this metabolic impairment in HFD-fed mice, as the HOMA index and non-fasting insulin normalized (Figures 2A,D) and the AUC and maximal decline in ITT were increased by TEL, while half-life was reduced (Figures 2F–IF). In contrast, TEL did not affect glucose control in chow-fed mice (Figure 2). The HOMA index positively correlated with plasma levels of ceramides and resistin (Figures 2B,C).

### Mucus Characteristics

**FIGURE 3:**
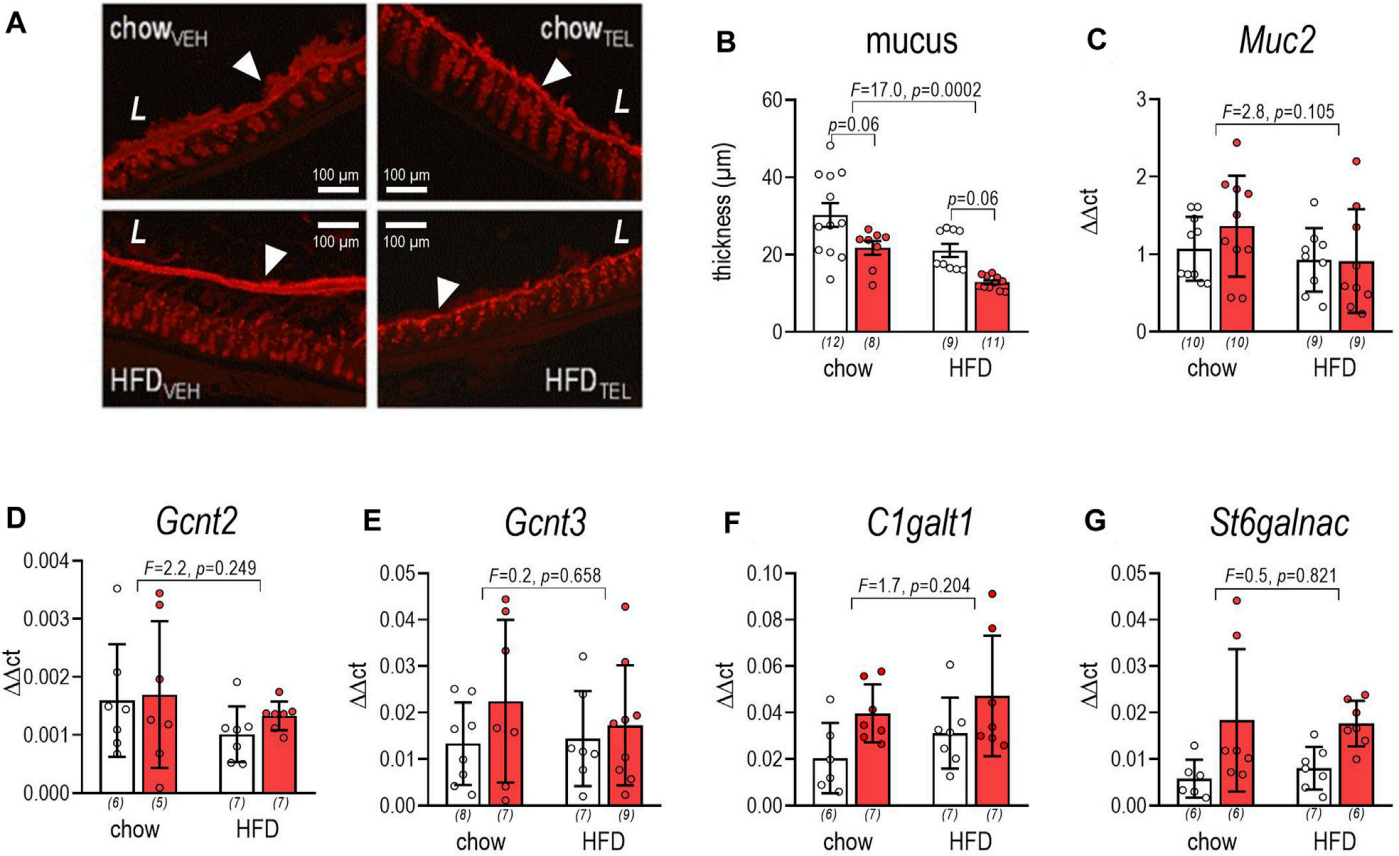
Effect of TEL (●, 8 mg/kg/day red bars) or vehicle treatment open bars (O) on mucus thickness of large intestine of mice that were fed either with chow or high fat diet (HFD). **(A)** staining against mucin-2 (arrows indicate mucus, L indicates the intestine lumen); **(B)** thickness of mucus; thickness was quantified using ZEN 2.3 imaging software by 40-fold multiple measurement per animal in different sections (for further details see also Supplementary Figure S4); two-way ANOVA; TEL: *F* = 20.1, *p* < 0.0001); **(C)** αmucin-2 expression (two-way ANOVA; TEL: *F* = 0.6, *p* = 0.441); **(D)** *Gcnt2* expression (two-way ANOVA; TEL: *F* = 0.4, *p* = 0.517); **(E)** Gcnt3 expression (two-way ANOVA; TEL: *F* = 1.7, *p* = 0.202); **(F)** *c1galt1* expression (two-way ANOVA; TEL: *F* = 6.4, *p* = 0.018); **(G)** *St6galnac* expression (two-way ANOVA; TEL: *F* = 10.8, *p* = 0.003). Means ± SD; the individual group size is indicated below the bars in brackets.

**FIGURE 4:**
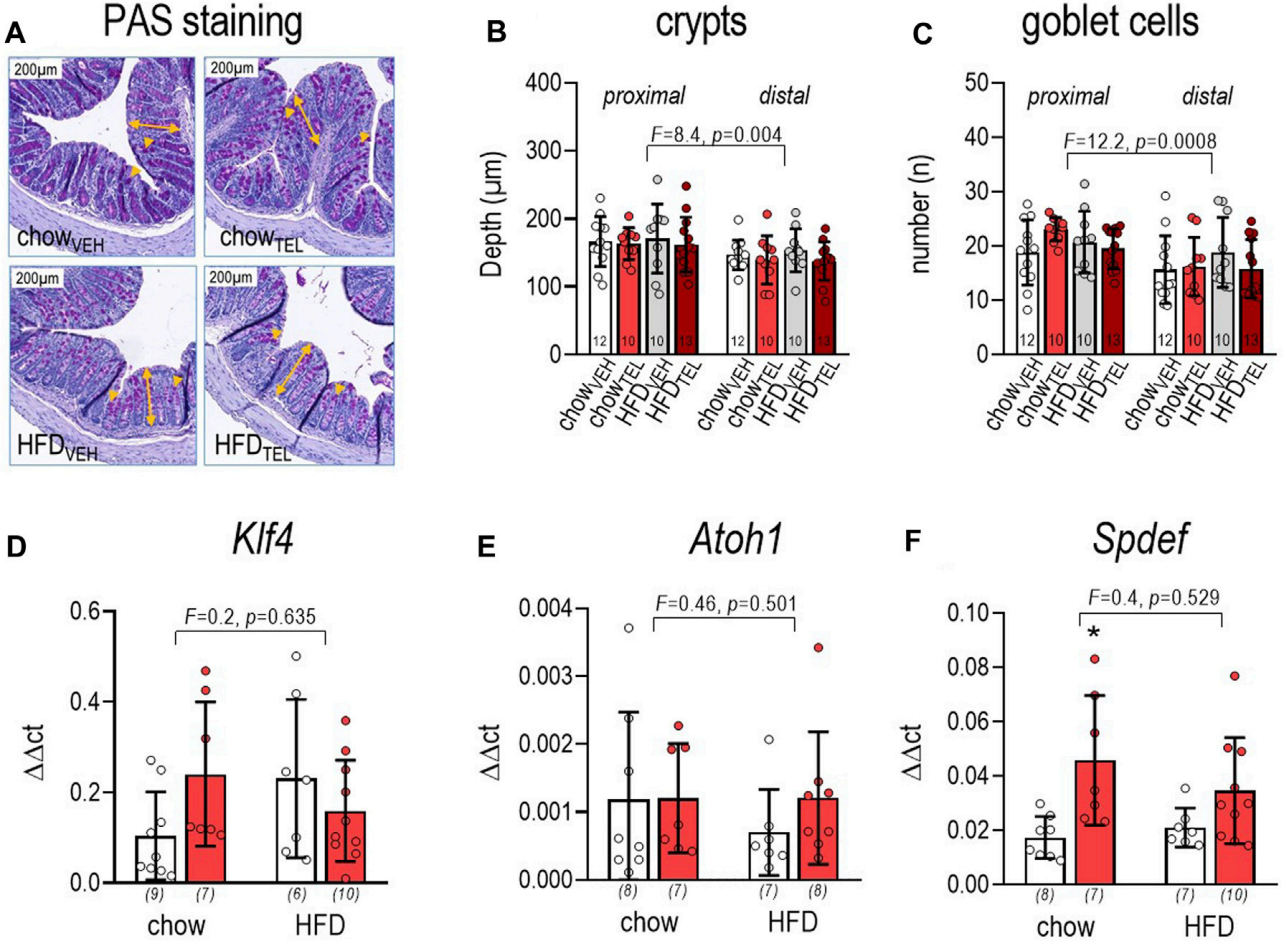
Effect of TEL (●, 8 mg/kg/day red bars) or vehicle treatment open bars (O) on large intestine of mice that were fed either with chow or high fat diet (HFD). **(A)** PAS staining, the yellow double arrows indicate depth of crypt, while yellow arrows indicate goblet cells depths of crypts were only measured in fully visible symmetric crypts; **(B)** depth of crypts, the respective crypt depth was measured in 10 defined crypts per image by considering four images per animal (three-way ANOVA; diet: *F* = 0.1, *p* = 0.774; TEL: *F* = 1.4, *p* = 0.239); **(C)** number of goblet cells; number of goblet cells within one crypt was counted in 10 crypts per image (three-way ANOVA; diet: *F* = 0.1, *p* = 0.795; TEL: *F* = 0.1, *p* = 0.882); **(D)** klf4 expression (two-way ANOVA; TEL: *F* = 0.5, *p* = 0.49); **(E)** atoh1 expression (two-way ANOVA; TEL: *F* = 0.54, *p* = 0.467); **(F)** *Spdef* expression (two-way ANOVA; TEL: *F* = 12.7, *p* = 0.001); means ± SD; the individual group size is indexed below the bars in brackets;.*p < 0.05 vs. chow_VEH_.

Staining against mucin-2 allowed us to quantify mucus thickness (Figure 3A). This was markedly lower in HFD- compared to chow-fed mice and, if at all, slightly (*p* = 0.06) lowered in both diet groups by TEL (Figure 3B). However, expression of *Mucin-2* was not altered in relation to diet or TEL (Figure 3C). To characterize mucus biosynthesis in more detail, we further determined expression of 6-N-acetylglucosaminyl-transferase *Gcht2* and *Gcht3*, β1,3-galactosyltransferase (*C1galt1*), and α2,6-sialytransferase (*St6galnac*), which all facilitate O-glycosylation of mucin-2. None of these markers was influenced by diet (Figures 3D–G). However, two-way ANOVA indicated that *St6galnac* (*F* = 10.8, *p* = 0.003) and *C1galt1* (*F* = 6.4, *p* = 0.018) increased in response to TEL. Applying PAS staining (Figure 4A), we found that the depth of crypts and amount of goblet cells were lower in distal versus proximal segments of the large intestine, but neither diet nor TEL affected these parameters (Figures 4B,C). As *Klf4*, *Atoh1*, and *Spdef1* are involved in the differentiation of goblet cells, expression of these factors was determined by qPCR. However, none of these markers was influenced by diet (Figures 4D–F). *Spdef1* increased in response to TEL (*F* = 12.7, *p* = 0.001, Figure 4F). Using H&E staining and measuring fecal albumin, we respectively detected that no group showed signs of intestine inflammation (Supplementary Figure S5A) or intestinal barrier dysfunction (Supplementary Figure S5B).

**FIGURE 5:**
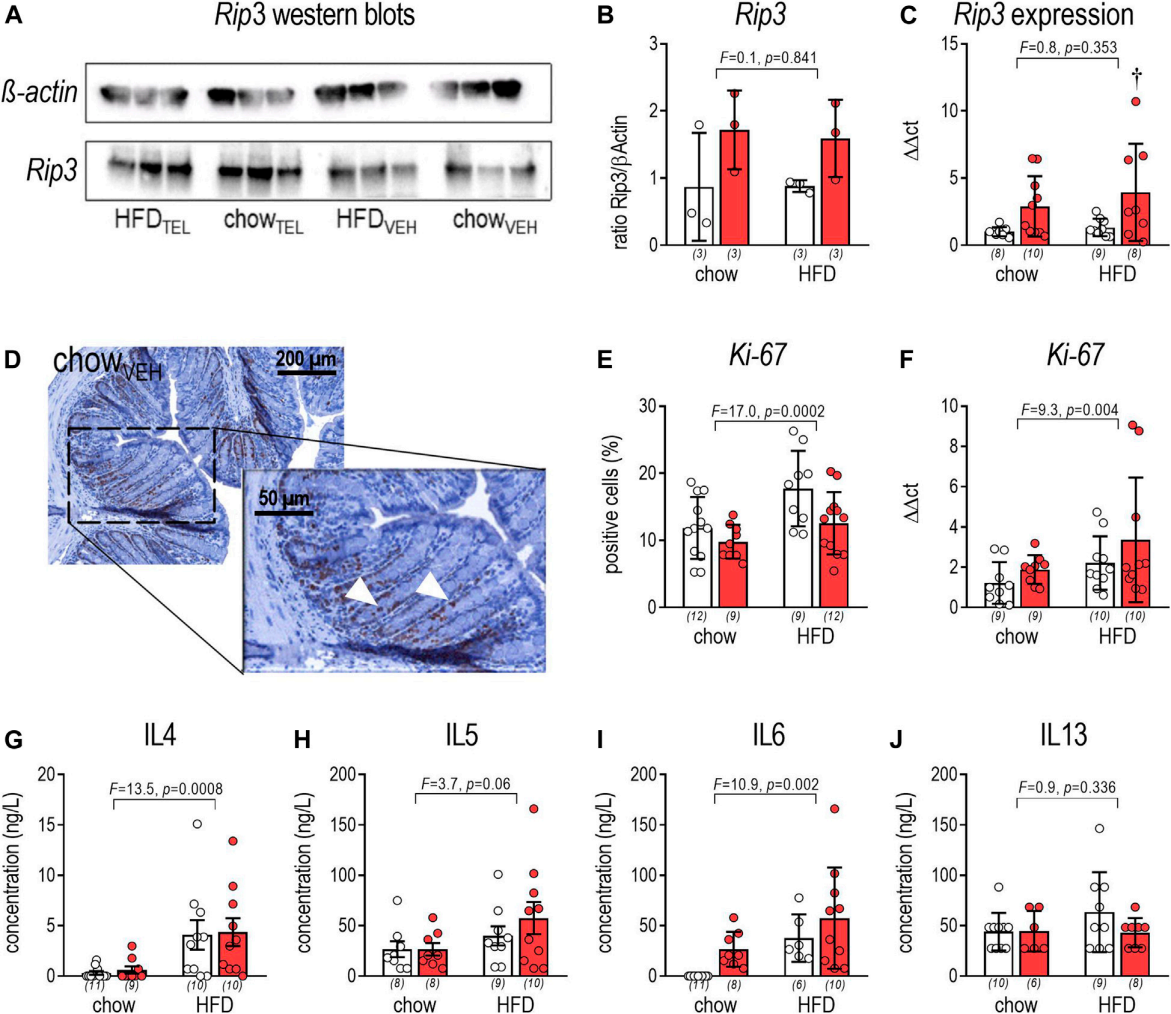
Effects of TEL (●, 8 mg/kg/day red bars) or vehicle treatment open bars (O) on large intestine on necroptosis, proliferation, and inflammation. **(A)** *Rip3* western blots; **(B)** *Rip3/β-actin* ratio (two-way ANOVA; TEL: *F* = 5.5, *p* = 0.047, interaction; *F* = 0.1, *p* = 0.841); **(C)** *Rip3* expression in large intestine (two-way ANOVA; TEL: *F* = 9.6, *p* = 0.004, interaction; *F* = 0.3, *p* = 0.616); **(D)** staining against *Ki-67 in vehicle treated chow-fed controls; Ki-67 positive cells are indicated by an arrow.*; **(E)** *Ki-67*-positive cells (Staining was evaluated using ImageJ (version 2.0.0-RC-54/1.51 h), in which 4 areas of interst were defined for each animal, with the same number of pixels for each image. Within this area, the percentage of Ki-67 positive cells was measured, averaged, and expressed as a percentage based on the Ki-67 labeling index; two-way ANOVA; TEL: *F* = 20.1, *p* < 0.0001, interaction; *F* = 2.9, *p* = 0.097); **(F)** *Ki-67* expression (two-way ANOVA; TEL: *F* = 6.6, *p* = 0.0146, interaction; *F* = 1.2, *p* = 0.278); plasma levels after 12 weeks of IL4 (G; two-way ANOVA; TEL: *F* = 0.1, *p* = 0.785, interaction; *F* < 0.01, *p* = 0.991); IL5 (H, two-way ANOVA; TEL: *F* = 0.6, *p* = 0.445, interaction; *F =* 0.06, *p* = 0444); IL6 (I; two-way ANOVA; TEL: *F* = 5.0, *p* = 0.032, interaction; *F* = 0.1, *p* = 0.739); IL13 (J; two-way ANOVA; TEL: *F* = 1.2, *p* = 0.287, interaction; *F* = 1.3, *p* = 0.256). Means ± SD, †*p* < 0.05 vs. HFD_VEH_. The individual group size is indicated below the bars in brackets.

*Rip3*, which is a key player in the necrosis signaling pathway (Moriwaki and Chan, 2013), was shown in western blots and qPCR analyses to not be affected by diet but to be increased by TEL (Figures 5A–C). We further determined *Ki-67* as a proliferation marker to visualize rapidly dividing cell populations. By staining against *Ki-67*, *Ki-67*-positive cells were higher in HFD- than chow-fed mice (two-way ANOVA: *F* = 17.0, *p* = 0.0002), but lowered in both diet groups by TEL (two-way ANOVA: *F* = 20.1, *p* < 0.0001) without reaching significance levels <0.05 in multiple post hoc testing via Sidak’s multiple comparisons test (Figures 5D,E). The diet effect on *Ki-67* was confirmed by qPCR analyses (Figure 5F). In contrast to the *Ki-67* staining experiments, TEL was not found to lower *Ki-67* expression (Figure 4F). We further determined circulating cytokines by the microsphere-based multiplex technique. Plasma levels of interleukin (IL)-4 and IL-6 of chow-fed mice were within the range of the detection limit. In response to HFD feeding, IL-4, IL-5, and IL-10 markedly rose in both diet groups. IL-6 rose in TEL-treated animals (Figures 5G–I). IL-13 was found to be regulated neither by diet nor by TEL (Figure 5J). Other cytokines were found below the detection limit.

**FIGURE 6:**
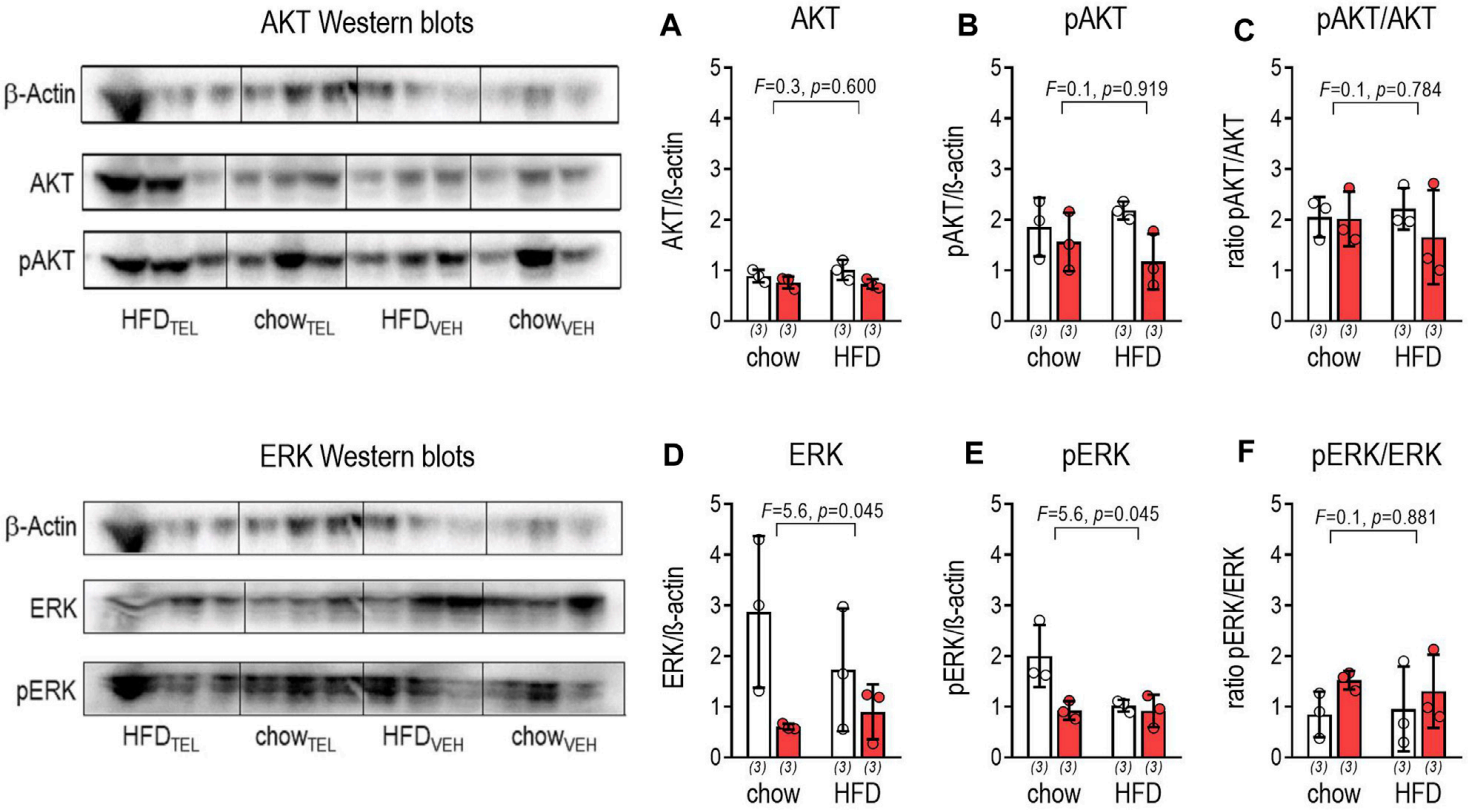
Effects of TEL (●, 8 mg/kg/day red bars) or vehicle treatment open bars (O) on protein expression of AKT and ERK on large intestine of chow- or HFD-fed mice. **(A)** AKT/β-actin ratio (two-way ANOVA; TEL: *F* = 6.3, *p* = 0.037; interaction; *F* = 0.8, *p* = 0.386); **(B)** pAKT/β-actin ratio (two-way ANOVA; TEL: *F* = 5,1, *p* = 0.054; interaction; *F* = 0.6, *p* = 0.761); **(C)** pAKT/AKT ratio (two-way ANOVA; TEL: *F* = 0.7, *p* = 0.418; interaction; *F* = 0.5, *p* = 0.476); **(D)** ERK/β-actin ratio (two-way ANOVA; TEL: *F* = 7.2, *p* = 0.028, interaction; *F* = 1.5, *p* = 0.249); **(E)** pERK/β-actin ratio (two-way ANOVA; TEL: *F* = 7.9, *p* = 0.0027, interaction; *F* = 5.4, *p* = 0.049); **(F)** pERK/ERK ratio (two-way ANOVA; TEL: *F* = 2.2, *p* = 0.180, interaction; *F* = 0.2, *p* = 0.654). Means ± SD, means ± SD (n = 3).

*AT*_*1A*_ *, AT*_*1B*_ *, and AT*_*2*_ receptors were found by PCR to be expressed in large intestine of mice below the detection limit (Supplementary Figure S5). The intracellular AKT pathway was not affected by diet. However, TEL reduced AKT but not phosphorylated AKT (pAKT) (Figures 6A–C). The extracellular signal-regulated kinase 1/2 (ERK) pathway was influenced by diet and TEL as both ERK and ERK phosphorylation (pERK) were reduced (Figures 6D,E). However, the pERK/ERK ratio itself was not significantly enhanced, thus indicating that TEL did not stimulate the ERK pathway (Figure 6F).

### *In-vitro* Experiments to Investigate Cell Viability

**FIGURE 7:**
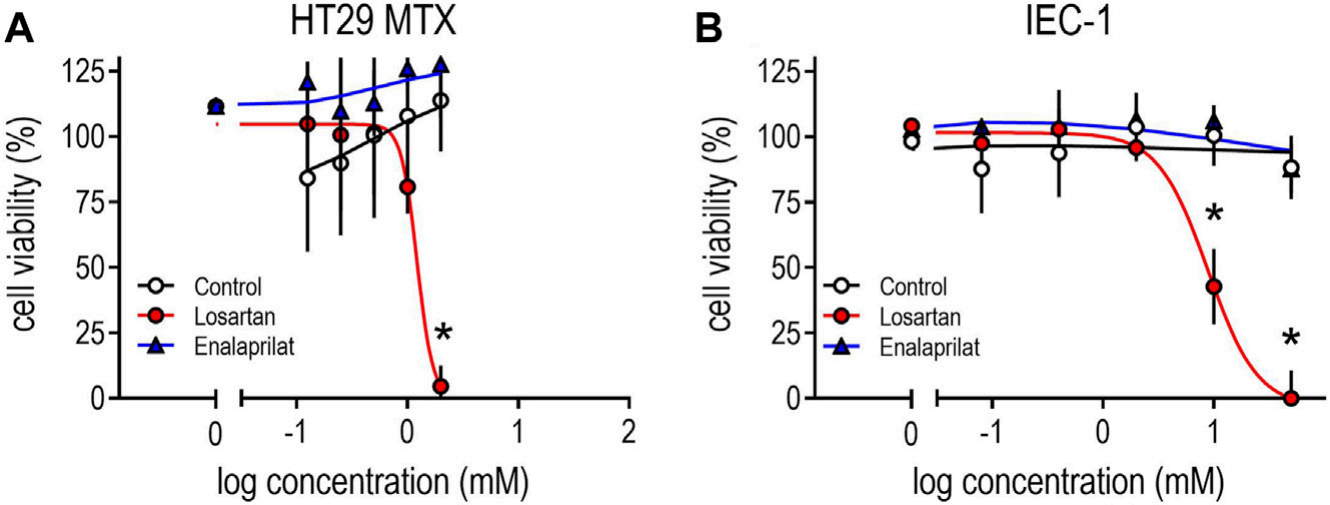
Effects of losartan or enalaprilat on cell viability in mucus-producing HT29-MTX cells **(A)**; (IC_50_ 1.2 mM) or in murine intestine epithelium cells **(B)**; (IEC-1; IC_50_ 8.8 mM). TritonX100 (0.5%) served as a control for total cell death. Cell viability was measured by using a commercial MTS assay after incubating cells for 8 days (HT29-MTX) or 3 days (IEC-1) with losartan (0.08–50 mM) or enalaprilat (0.1125–2 mM). Means ± SD; (n = 5), *p < 0.05 vs. corresponding controls.

Finally, we performed cell culture experiments by using the mucus-producing cell line HT29MTX or small IEC to investigate cell viability in the presence of the ARB losartan and the ACE inhibitor enalaprilat. Increasing concentrations of losartan induced cell death in both cell lines: at concentrations of 2 mM losartan, cell viability in HT29MTX was comparable to the effects of the cell dead control (Figure 7A). Losartan also reduced cell viability in IEC at concentrations of 10 and 50 mM (IC_50_ = 8.3 mM) to a comparable extent to that of cell dead controls (Figure 7B). Equivalent treatments with enalaprilat did not affect cell growth in either cell line (Figure 7A).

## Discussion

In accordance with previous studies in mice and rats (Muller-Fielitz et al., 2011; Miesel et al., 2012; Muller-Fielitz et al., 2012; Muller-Fielitz et al., 2014; Muller-Fielitz et al., 2015; Schuchard et al., 2015; Schuster et al., 2018; Winkler et al., 2018; Gustaityte et al., 2019; Rawish et al., 2020; Huber et al., 2021), we were able to show here that, on the one hand, HFD feeding leads to the development of obesity, leptin and insulin resistance, as well as hyperlipidemia, while on the other hand, treatment with TEL can prevent these scenarios. In this context and for example, a leptin- (Muller-Fielitz et al., 2011; Muller-Fielitz et al., 2012; Schuster et al., 2018), ACE2/Ang (1–7)/Mas axis- (Blanke et al., 2015; Schuchard et al., 2015; Dapper et al., 2019), and brain-related mechanism (Winkler et al., 2016; Huber et al., 2021) has been investigated and discussed as a cause for the anti-obese potency of TEL. For the sake of brevity, we refrain from such a detailed discussion here and refer to the corresponding publications. We intended here to follow-up on our recently published data showing that TEL induces a specific gut microbiota signature that may mediate its anti-obesity effect (Beckmann et al., 2021). Since HFDs damage endothelial barrier properties by alterations in microbiota (Araújo et al., 2017), the aim of the present study was to investigate whether TEL has protective effects with respect to endothelial barrier function.

### Reduction of Mucus Thickness Upon HFD Feeding

HFD induces intestinal dysbiosis in intervillous spaces and crypts, which is associated with early pathophysiological changes, predominantly in the ileum, such as low-grade inflammation, decreased antimicrobial peptide expression, impaired mucus production, secretion, and layer thickness, as well as decreased expression of tight junction proteins (Araújo et al., 2017). Although we did not investigate the gastrointestinal microbiome in this study, we have previously demonstrated intestinal changes in composition balance in mice under the HFD also used here (Beckmann et al., 2021).

While mucus thickness was lower, *Muc2* expression was only tendentially reduced in response to HFD feeding, and albumin excretion (as a sign of worsened intestinal permeability) was unchanged. These findings partially confirmed others who showed that feeding mice with a high-fat and high-sugar diet decreases mucus layer thickness, probably due to lower *Muc2* gene expression, and increases intestinal permeability (Martinez-Medina et al., 2014). Loss in mucus thickness has been postulated, on the one hand, to be related to a lower intake of dietary fiber when mice received HFD, thus enhancing the use of mucus glycoproteins as a nutrient source for intestinal bacteria (Desai et al., 2016). On the other hand, the mucin secretion capacity of the mucosa is related to the number of goblet cells in the villi and/or crypts in the gut. Hence, long-term treatment of mice with nicotinamide mononucleotide increased the number of goblet cells and promoted mucus secretion (Huang et al., 2021), whereas conversely, the number of goblet cells and mucin were reduced in rats suffering from colitis (Pélissier et al., 2006) or in HFD-fed mice (Huang et al., 2021). However, we did not detect any effect on the number of goblet cells in response to HFD feeding. Thus, our results differ from those of Baldassano et al. (Baldassano et al., 2013) who found an increase in cell numbers per villus even after 14 weeks on an HFD. Moreover, we did not detect any effect on crypt depth, while Baldassano et al. detected an increase in crypt-villus (Baldassano et al., 2013), which prompted them to corroborate the hypothesis that in obese mice, the increased surface of absorption contributes to the weight gain of these animals (De Wit et al., 2008). Based on our results, we can neither confirm nor refute this hypothesis, concluding instead that the reduction in mucus thickness was more likely due to degradation of glycoproteins by intestinal bacteria (e.g., *Akkermansia muciniphila, Bacteroides caccae, Bacteroides ovatus, and Eubacterium rectale*), as postulated by others (Desai et al., 2016). Consequently, the intestinal abundance of such mucin O-glycan degraders in the feces of animals should be investigated in follow-up studies.

To further assess the dynamic processes between synthesis and erosion of mucin, we determined *Ki-67*-positive cells and *Ki-67* expression in tissue samples, as *Ki-67* serves as a marker for cell proliferation as well as *Rip3* protein levels, as *Rip3* is a key component in the signaling pathway of necroptosis. Here we found that the number of *Ki-67*-positive cells and *Ki-67* expression are higher in intestine of HFD-fed mice, which is now in agreement with Baldassano et al., who also found that the *Ki-67*-positive cell number in the crypt region was enhanced in HFD-fed compared to chow-fed mice (Baldassano et al., 2013). However, *Rip3* protein and mRNA levels remained unchanged, thereby suggesting that the unchanged number of goblet cells is more likely due to a dynamic balance between proliferation and necrosis. Since we did not detect an altered number of goblet cells, but reduced mucus thickness, we next asked whether factors that play an important role in regulating mucus production might provide clues as to whether reduced mucus thickness could be related to a regulation of such expression factors. 6-N-acetylglucosaminyl-transferase (Gcnt2 and Gcnt3), β1,3-Galactosyltransferase (*C1galt1*), and α2,6-sialytransferase (*St6galnac*) all facilitate O-glycosylation of mucins (Bergstrom and Xia, 2013). *Spdef* regulates intestinal epithelial cell homeostasis and differentiation, as loss of *Spdef* impairs maturation, while expression of *Spdef* promotes goblet cell differentiation (Gregorieff et al., 2009; Noah et al., 2010). The transcription factor atonal homolog 1 (Atoh1) plays a critical role in secretory cell differentiation (Vandussen and Samuelson, 2010). Nevertheless, we obtained no evidence from our qPCR measurements that any of these factors is decreased in large intestine of HFD-fed mice.

We further asked whether the ERK- or AKT-related pathway may be involved in HFD-related reduction of mucus thickness. The phosphorylation of ERK leads to the activation of transcription factors and, thus, to the expression of genes that regulate the proliferation and survival of cells (Steelman et al., 2011). Using different stimuli, ERK1/2 activation was demonstrated *in vitro* to contribute to *Muc2* secretion from goblet cells (Lee et al., 2002; Lee et al., 2010; Damiano et al., 2018; Kim et al., 2018). In addition, other substances have been found to upregulate mucin secretion of intestinal goblet cells via AKT phosphorylation (Wang et al., 2014; Yasuda-Onozawa et al., 2017). As intestine pERK, but not pAKT protein, as well as mucus thickness and, generally speaking, *Muc2* expression were lower upon HFD feeding, we conclude that the decrease in mucus secretion is related to the ERK rather than the AKT pathway. However, we concede that the group size of n = 3 in the Western blot analysis is small and thus the strength of evidence regarding AKT and ERK expression is somewhat limited.

We finally asked whether HFD-induced changes in various plasma parameters are correlated or perhaps even causal for alterations in mucus. In this regard, it has been shown in experimental mouse models of inflammatory bowel disease that increased intestinal IL-6 tissue levels correlate with decreased mucus thickness (Nguyen et al., 2015; Ahl et al., 2016). This, in turn, appears to be consistent with the increased plasma IL-6 levels observed here in mice with HFD compared with chow diet, suggesting inflammatory causality for the decreased mucus thickness. Moreover, others have shown a number of protective effects, such as: 1) Insulin stimulates production of glycoconjugate layers on the cell surface of a gastric mucous cell line (Tabuchi et al., 1997); 2) triglycerides enhanced mucus barriers (Yildiz et al., 2015); and 3) adiponectin prevents goblet cell apoptosis and increases goblet cell differentiation (Saxena et al., 2013). Only the abovementioned findings on triglycerides could be confirmed by this study since both mucus thickness and triglycerides were reduced under HFD. In addition, we have shown here and elsewhere increased insulin and adiponectin plasma concentrations under HFD (Muller-Fielitz et al., 2012; Muller-Fielitz et al., 2014), which should be protective on the mucus according to the cited studies (Tabuchi et al., 1997; Saxena et al., 2013). However, since we observed decreased mucus thickness in this study despite increased insulin levels under HFD, we tend to believe that these effects are of minor relevance in the setup of our study.

### Influence of TEL on Mucus Thickness

Based on the afore discussed findings that HFD-induced intestinal dysbiosis is associated with impaired mucus production, secretion, and layer thickness (Araújo et al., 2017), as well as on our recent findings that TEL beneficially influences GUT microbiota and obesity (Beckmann et al., 2021), we consequently investigated whether HFD-induced impairment of mucus is antagonized by TEL. In contrast to our expectation, mucus thickness was reduced rather than enhanced, thus suggesting an impaired gastric mucosal barrier. Indeed, the ARB olmesartan increased both the risk for enteropathy and lymphocytosis and the eosinophil count in duodenal mucosa of patients, all together indicating inflammatory processes. These observations led the authors to speculate that weight loss in olmesartan-treated patients is related to malabsorption and a higher risk for acute diarrhea (Costetti et al., 2021). However, albumin excretion was not impaired in our study, and diarrhea has never been identified in the past in rats or mice when TEL was given even in high doses (Muller-Fielitz et al., 2011; Muller-Fielitz et al., 2012; Muller-Fielitz et al., 2014; Muller-Fielitz et al., 2015; Schuchard et al., 2015; Winkler et al., 2016; Schuster et al., 2018; Dapper et al., 2019; Gustaityte et al., 2019; Rawish et al., 2020; Beckmann et al., 2021; Huber et al., 2021). Thus, our findings tend to confirm the data of Malfertheiner et al. (Malfertheiner et al., 2018), who found that ARBs indeed induced ("Sprue-like enteropathy") but to a much lesser extent, as observed by others (Rubio-Tapia et al., 2012; Costetti et al., 2021).

We also cannot confirm the observations on inflammatory effects under ARBs (Costetti et al., 2021). Even though we did not measure intestinal cytokine levels, plasma levels of cytokines in plasma (serving as a surrogate parameter) remained unchanged, except of the pro-inflammatory cytokine IL-6 (Schett, 2018). This increase in IL-6 upon TEL treatment was unexpected, given that other investigators have demonstrated reduced IL-6 plasma levels in response to ARBs in cells (Skurk et al., 2004; Iwashita et al., 2012) and in humans or animals suffering from cardiovascular or metabolic diseases (Schieffer et al., 2004; Toblli et al., 2008), thus suggesting anti-inflammatory effects under ARBs. The reason for this unexpected increase is unclear, particularly as we recently found in mice using similar feeding and treatment regimes that pro-inflammatory cytokines were downregulated upon TEL in adipose tissue or brain (Schuster et al., 2018; Rawish et al., 2020). Various reviews additionally confirm the notion of an anti-inflammatory potency of ARBs especially in cerebral (Saavedra, 2012), renal (Saldanha Da Silva et al., 2017), metabolic (Ferder et al., 2006), and vascular disorders (Wassmann and Nickenig, 2006). Thus, it is a limitation of our study that we did not measure intestinal cytokine levels, which should be considered in follow-up studies.

The opposite result to that expected, namely increased rather than decreased mucus thickness, as seen here upon TEL, raises the question of why. Less *Ki-67*-positive cells and more *Rip3* protein, which was also observed under TEL treatment, clearly indicates a reduction of proliferation and an increase of necrosis, thus probably reducing mucus thickness. Next, to verify whether ARBs have a direct cell toxic effect, cell viability was determined *in vitro* using mucus-producing HT29MTX cells or intestine endothelium cells. As mentioned above, the limited water solubility of TEL justified the use of LOS in the cell culture experiments. Indeed, LOS reduced viability in both cell lines when using a concentration higher than 1 mM. Considering TEL’s molecular mass of 515, a dose of 8 mg/kg_bw_ for mice experiments, and an application volume of 5 µl/g_bw_, the bolus concentration of the daily TEL dose was extrapolated to be 3 mM. Cell toxic effects were observed in both cell lines at concentrations >1 mM. Nevertheless, also considering dilution during gastrointestinal passage, it seems more likely that local intestinal TEL concentrations were markedly below 1 mM, thereby excluding direct toxic effects. Therefore, to further assess potential toxic effects of ARBs, *in vitro* cell experiments with the ACE inhibitor enalaprilat (ENA) were additionally performed, as the development of SLE has recently also been described for this substance group, albeit to a much lesser extent (Malfertheiner et al., 2018). Interestingly, cell viability remained unaffected under equal concentrations of ENA. These results are consistent with clinical findings showing that SLE occurred as a result of exposure to the class of ARBs and is not related to a general blockade of the RAS (Malfertheiner et al., 2018). Based on the results of the present study, it can be discussed whether the reduction in cell viability observed under LOS is due to the above-described RIP3-dependent form of cell death under ARB therapy and thus offers an explanatory approach for the pathogenesis of SLE, as seen in clinical trials (Rubio-Tapia et al., 2012; Malfertheiner et al., 2018; Costetti et al., 2021). In this regard, further complex studies are needed to fully evaluate the local toxic effects of ARBs on gastrointestinal barrier cells. Such studies may include functional experiments in addition to fecal albumin level, such as the fluorescein isothiocyanate-dextran-4 assay to assess intestinal barrier (Yu et al., 2020).

In addition to findings more suggestive for cell death, we have also obtained results that tend to indicate the opposite. Treatment with TEL increased the expression of St6galnac and thus the proportion of sialylated Tn antigen no longer participating in MUC2 structure formation. Hypersialylation is associated with harmful effects, as cancer cells typically exhibit hypersialylation, which contributes to cancer cell progression and metastasis (Wang et al., 2016). These results may provide another explanation for the reduction in the mucosal layer induced by TEL. Interestingly, treatment with TEL also showed upregulation of c1galt1 and Gcnt3. Both glycosyltransferases are essential for the expansion and maintenance of the MUC2 structure (Bergstrom and Xia, 2013). It could be hypothesized that there is a reflex increase in “core”-forming glycosyltransferases due to the decreased substrate supply in the context of hypersialylation. Treatment with TEL had no effect on β1,6-N-acetylglucosaminyltransferase encoded by Gcnt2. In agreement with these results, spdef [influencing goblet cell maturation (Gregorieff et al., 2009)] was found to be upregulated under TEL in the present study, which could also indicate a reflexive stimulus to compensate for the reduced mucus layer.

Finally, we address the question of whether the effects of TEL on the mucus observed here are attributed to an AT_1_R- or PPARγ-dependent mechanism, as it is well established that TEL not only blocks AT_1_R but also activates PPARγ (Doggrell, 2004; Erbe et al., 2006). Although PPARγ plays a dominant role in adipose tissue, it is also expressed in colonic epithelia (Lefebvre et al., 1999). *In vitro* studies have shown that PPARγ can suppress inflammatory responses by limiting the production of cytokines (Jiang et al., 1998). From a pathophysiological point of view, PPARγ is downregulated in colonic epithelial cells from ulcerative colitis patients (Dubuquoy et al., 2003). Hence, PPARγ agonists can attenuate colitis in mouse models (Su et al., 1999; Desreumaux et al., 2001). Moreover, PPARγ agonists increase the number of goblet cells, the glycosylation of mucins, and mucin2 expression (Imchen et al., 2013), thus indicating that PPARγ activation could promote the maturity and secretory function of goblet cells *in vivo* and thereby help maintain the integrity of the mucus layer. Hence, if the PPARγ-related effects of TEL were strong, we would have observed a protective effect on the mucus in the presence of TEL, which was certainly not the case. Consequently, this mechanism is comparatively unlikely. That PPARγ-associated effects do not necessarily occur *in vivo* (even though such effects might seem conceivable or probable) has already been shown in another context, as improved glucose tolerance upon TEL treatment, for example, was indeed not attributed to a PPARγ-dependent mechanism (Muller-Fielitz et al., 2012).

As a PPARγ-associated mechanism seems less likely, we have to consider an AT_1_R-dependent mechanism. Here, we failed to detect any mRNA levels of AT_1_R and AT_2_R in large intestines of mice, which, however, is not attributed to non-functional primer, as sufficient signals were obtained in adrenals. Despite our negative results and considering the findings of others detecting AngII receptors by the use of autoradiography and immunoreactivity in the intestine (Fändriks, 2011), the RAS is functionally established in the gastrointestinal tract. Particularly during blood volume and/or sodium depletion, AngII affects intestinal net fluid absorption and bicarbonate secretion in an AT_1_R-dependent manner (Fändriks, 2011). Thus, it might be speculated whether the water content within the mucus is reduced by TEL, thereby causing loss in mucus thickness. Supporting such a speculation, stimulation of AT_1_R was found to inhibit ion transport and to increase epithelial electrical resistance in esophageal mucosae, thus causing reduced paracellular permeability (Casselbrant et al., 2009). In addition to the direct antagonistic effect at AT_1_R, there is also evidence that TEL acts indirectly via the ACE2/Ang (1–7)/Mas axis (Schuchard et al., 2015). This alternative RAS axis reveals beneficial gastric effects as well as anti-metabolic, anti-inflammatory, and anti-proliferative effects (Singer and Camargo, 2011; Perlot and Penninger, 2013). Moreover, ACE2 was recently found to contribute to the proliferation of intestinal stem cells, thereby orchestrating mucosal homeostasis (Yu et al., 2020). Thus, it might be speculated whether a Mas-dependent mechanism participates in TEL actions observed here. Such an idea is supported by recent findings (Oliveira et al., 2020) showing an increase in mucus thickness and expression of *Ki-67* in Mas-knockout (ko) mice, thus fitting our observation that mucus thickness was lower and *Ki-67* expression was downregulated upon TEL treatment. Moreover, the B/F ratio was lowered in Mas-ko compared to wild-type (wt) mice (Oliveira et al., 2020), as well as in TEL-treated and CD-fed rats compared to controls only receiving CD (Beckmann et al., 2021). However, the Santos study (Oliveira et al., 2020) reveals certain inconsistencies with other findings in the literature. Body weight and fat mass were lower in Mas-ko compared to wt mice, which is on the one hand the opposite of other findings of the same group showing higher body weight and fat mass, as well as dyslipidemia and higher levels of insulin and leptin in Mas-ko mice (Santos et al., 2008). On the other hand, the development of diet-induced obesity was prevented by treating Sprague Dawley rats when the Mas receptor was stimulated with oral Ang (1–7), or in transgenic rats overexpressing Ang (1–7) (Santos et al., 2014; Blanke et al., 2015; Schuchard et al., 2015). Thus, even if a Mas-dependent mechanism seems possible, some unanswered questions remain, especially regarding the discrepant findings on the weight development of the Mas-deficient mice.

In summary, we have demonstrated here that mucus thickness decreased upon TEL and that *Ki-67* as a proliferation marker decreased in parallel, while *Rip3* as a necroptosis marker increased, thus supporting the finding on mucus histology. Follow-up studies are needed to elucidate whether this detrimental TEL effect is relevant for gastrointestinal function.

## Data Availability

The original contributions presented in the study are included in the article/**Supplementary Material**, further inquiries can be directed to the corresponding author.
