# Supplementary material for: The AT1 Receptor Blocker Telmisartan Reduces Intestinal Mucus Thickness in Obese Mice
Source: Front Pharmacol. 2022 Mar 31;13:815353. doi: 10.3389/fphar.2022.815353 (PMC9009210; doi:10.3389/fphar.2022.815353)
Supplement: Supplementary file 1 [file DataSheet1.docx]

Supplemental information to

The AT_1_ receptor blocker telmisartan reduces intestinal mucus thickness in obese mice

Laura Nickel^1,2^, Annika Sünderhauf^4^, Elias Rawish^1,2^, Ines Stölting^1^, Stefanie Derer^4^, Christoph Thorns^5^, Urte Matschl^6^, Alaa Othman^3.7^, Christian Sina^4^, Walter Raasch^1,2,3^

^1^Institute of Experimental and Clinical Pharmacology and Toxicology, University of Lübeck, Germany, ^2^DZHK (German Centre for Cardiovascular Research), partner site Hamburg/Kiel/Lübeck, Lübeck, Germany, ^3^CBBM (Centre of Brain, Behaviour and Metabolism, University of Lübeck), ^4^Institute of Nutritional Medicine, University Hospital Schleswig-Holstein, Campus Lübeck, Germany, ^1^Institute of Pathology, University of Lübeck, Germany, ^6^Department Virus Immunology, Heinrich Pette Institute, Leibniz Institute for experimental Virology, Hamburg, Germany ^7^Institute for Clinical Chemistry, University Hospital Zürich

Table s1. ANOVA table of three-way ANOVA test of development of body weight, energy expenditure (EE), respiratory ratio (RER), energy intake (EI), locomotion, and drinking (for figures see Fig. s2)

|  | Time | TEL | Diet | Time x TEL | Time x diet | TEL x diet | Time x TEL x diet |
| --- | --- | --- | --- | --- | --- | --- | --- |
| Body weight | *F*=264 *p*<0.0001 | *F*=54.3 *p*<0.0001 | *F*=25.9 *p*<0.0001 | *F*=76.6 *p*<0.0001 | *F*=34.5 *p*<0.0001 | *F*=23.1 *p*<0.0001 | *F*=39.1 *p*<0.0001 |
| EE | *F*=16.5 *p*<0.0001 | *F*=1.7 *p*=0.200 | *F*=2.5 *p*=0.118 | *F*=1.5 *p*<0.0001 | *F*=1.79 *p*<0.0001 | *F*=0.21 *p*=0.650 | *F*=0.97 *p*=0.640 |
| RER | *F*=34.0 *p*<0.0001 | *F*=0.0 *p*=0.929 | *F*=347 *p*<0.0001 | *F*=1.9 *p*<0.0001 | *F*=12.9 *p*<0.0001 | *F*=0.45 *p*=0.505 | *F*=0.8 *p*=0.981 |
| EI | *F*=5.4 *p*<0.0001 | *F*=9.1 *p*=0.004 | *F*=90.1 *p*<0.0001 | *F*=0.96 *p*=0.660 | *F*=1.44 *p*<0.0001 | *F*=0.0 *p*=0.950 | *F*=1.28 *p*=0.036 |
| Locomotion | *F*=7.1 *p*<0.0001 | *F*=0.45 *p*=0.508 | *F*=0.04 *p*=0.829 | *F*=1.1 *p*=0.053 | *F*=0.95 *p*=0.715 | *F*=3.6 *p*=0.063 | *F*=1.23 *p*=0.003 |
| Drinking | *F*=8.5 *p*<0.0001 | *F*=28.3 *p*<0.0001 | *F*=16.3 *p*=0.0002 | *F*=1.4 *p*<0.0001 | *F*=1.8 *p*<0.0001 | *F*=1.8 *p*=0.185 | *F*=1.3 *p*=0.001 |

*TEL*, telmisartan

Figure s1: Study protocol

Figure s2: Weight regulation of mice that were fed either with chow or high fat diet (HFD). Mice were treated with TEL (●O , 8 mg/kg/day) or vehicle (O). A: exemplary MRI images; B: sub cutaneous fat mass (two-way ANOVA; TEL: *F*=20.1, *p*<0.0001, interaction; *F*=18.5, *p*=0.0005); C: visceral fat mass (two-way ANOVA; TEL: *F*=47.6, *p*<0.0001, interaction; *F*=21.7, *p*=0.0002); D: left ventricular weight (two-way ANOVA; TEL: *F*=163.1, *p*<0.0001, interaction; *F*=5.2, *p*<0.027); E: liver weight (two-way ANOVA; TEL: *F*=26.5, *p*<0.0001, interaction; *F*=12.1, *p*=0.0015); F: kidney weight (two-way ANOVA; TEL: *F*=30.0, *p*<0.0001, interaction; *F*=13.1, *p*=0.0008); means±SD, * p<0.05 vs. chow_VEH_, † p<0.05 vs. corresponding vehicle treatment

Figure s3: Energy homeostasis of mice that were fed either with chow or high fat diet (HFD). Mice were treated with TEL (8 mg/kg/day) or vehicle. A three-way ANOVA was calculated considering diet, TEL, and time (for results see Table s1). Means±SEM (n=13 chow_VEH_, n=13 HFD_VEH_, n=11chow_TEL_, n=12 HFD_TEL_)


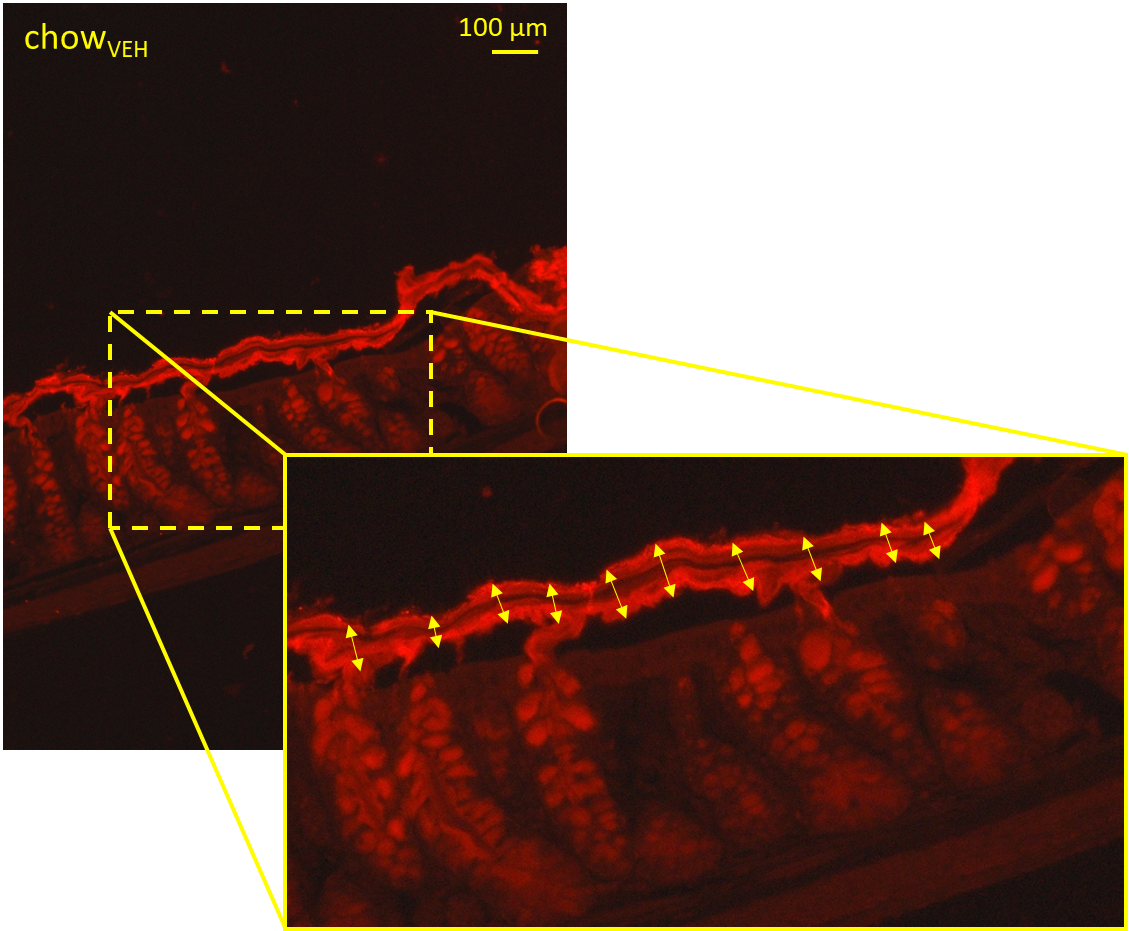


Figure s4: An example image of staining against mucin-2. Based on immunofluorescence microscopic images of this kind, a section of intact mucus was identified and, within this area, the thickness of the mucus measured (using ZEN 2.3 imaging software) by 10-fold determination (represented by the yellow double arrows). Within one animal, four such images were obtained, meaning that the evaluation was based on approximately 40 individual measurements per animal

Figure s5: Effect of TEL (●O , 8 mg/kg/day) or vehicle (O) on large intestine of mice that were fed either with chow or high fat diet (HFD). A: H&E staining; B: fecal albumin concentration (two-way ANOVA; TEL: *F*=0.1, *p=*0.934, interaction; *F*=0.6, *p*=0.457); means±SD

Figure s5: Effect of TEL (8 mg/kg/day) on expression of AT_1A_, AT_1B_, and AT_2_ receptors in large intestine of mice that were fed either with chow or high fat diet (HFD). Adrenal glands served as positive controls for AT receptor expression
